# Ultrasmall Solid-Lipid Nanoparticles via the Polysorbate Sorbitan Phase-Inversion Temperature Technique: A Promising Vehicle for Antioxidant Delivery into the Skin

**DOI:** 10.3390/pharmaceutics15071962

**Published:** 2023-07-16

**Authors:** Francesca Della Sala, Assunta Borzacchiello, Chiara Dianzani, Elisabetta Muntoni, Monica Argenziano, Maria Teresa Capucchio, Maria Carmen Valsania, Annalisa Bozza, Sara Garelli, Maria Di Muro, Franco Scorziello, Luigi Battaglia

**Affiliations:** 1 Institute of Polymers, Composites and Biomaterials, National Research Council (IPCB-CNR), Viale J.F. Kennedy 54, 80125 Naples, Italy; francesca.dellasala@cnr.it (F.D.S.); bassunta@unina.it (A.B.); 2 Dipartimento di Scienza e Tecnologia del Farmaco, University of Turin, Via Pietro Giuria 9, 10125 Turin, Italy; chiara.dianzani@unito.it (C.D.); elisabetta.muntoni@unito.it (E.M.); monica.argenziano@unito.it (M.A.); annalisa.bozza@unito.it (A.B.); saragarelli.sg@gmail.com (S.G.); 3 Department of Veterinary Sciences, University of Turin, Largo Paolo Braccini 2, 10095 Grugliasco, Italy; mariateresa.capucchio@unito.it; 4 Department of Chemistry, University of Turin, Via Quarello 15/a, 10135 Turin, Italy; mariacarmen.valsania@unito.it; 5 Nanostructured Interfaces and Surfaces (NIS) Interdepartmental Centre, 10124 Turin, Italy; 6 R Bio Transfer srl, Via Parmenide 156, 84131 Salerno, Italy; mar.dimuro@gmail.com (M.D.M.); studiofs@hotmail.com (F.S.)

**Keywords:** solid lipid nanoparticles, phase inversion, ultrasmall, skin, anti-age

## Abstract

Solid lipid nanoparticles promote skin hydration via stratum corneum occlusion, which prevents water loss by evaporation, and via the reinforcement of the skin’s lipid-film barrier, which occurs through the adhesion of the nanoparticles to the stratum corneum. The efficacy of both phenomena correlates with lower nanoparticle size and the increased skin permeation of loaded compounds. The so-called Polysorbate Sorbitan Phase-Inversion Temperature method has, therefore, been optimized in this experimental work, in order to engineer ultrasmall solid-lipid nanoparticles that were then loaded with α-tocopherol, as the anti-age ingredient for cosmetic application. Ultrasmall solid-lipid nanoparticles have been proven to be able to favor the skin absorption of loaded compounds via the aforementioned mechanisms.

## 1. Introduction

In healthy skin, the stratum corneum typically has a water content of 20% and provides an efficient barrier against the percutaneous absorption of exogenous substances. This barrier can be altered by skin occlusion, which increases stratum corneum hydration. In fact, solid-lipid nanoparticles (SLNs) promote skin hydration via two mechanisms: (1) the formation of an occlusive film on the surface of the stratum corneum, which prevents water loss by evaporation; (2) the reinforcement of the skin’s lipid-film barrier via nanoparticle adhesion to the stratum corneum. The greatest occlusion is achieved using low-melting-point lipids that are highly crystalline and have very small sized particles. Indeed, nanoparticles were found to be 15 times more occlusive than microparticles, while those smaller than 400 nm and that contain at least 35% lipids with a high crystallinity degree are the most effective [1]. The ability of SLNs to adhere to the stratum corneum is linked to their composition, since they can interact with it, modifying its lipid rearrangement and facilitating the penetration of molecules. Small nanoparticles contribute more significantly to the increased adhesiveness and surface contact [2].

Moreover, the augmented skin absorption of the compounds loaded inside SLNs may also result from increased contact surface with the corneocytes, from rapid or prolonged release and from the surfactants employed in the SLN suspension [3]. Indeed, SLN suspensions are stabilized by surfactants, which increase the skin penetration of the loaded ingredients as they alter the skin structure [2]. SLNs are also more advantageous than classic ointments, whose occlusion does not guarantee rapid hydration, especially if the stratum corneum is excessively dry, making a water supplement desirable. SLNs suspensions are suitable for this purpose because, when applied onto the skin, particle fusion forms a dense film, with this formation being promoted by the capillary forces involved during the evaporation of water [1].

Finally, although lipid nanoparticles do not generally penetrate through the stratum corneum, absorption by hair follicles has been reported. In particular, some studies have shown that entry via the follicular pathway is preferred by particles with smaller particle size [2]. In fact, the hair follicle mainly consists of a sebum-rich environment that is composed of lipids, waxes and squalene, and this facilitates the deeper penetration of lipid nanoparticles. The subsequent release of active ingredients is attributed to the erosion or dissolution of the lipid matrix in the sebum, thus favoring the deeper percutaneous absorption of the loaded ingredients [4].

Cosmetic ingredients should not be absorbed systemically, but some penetration into the skin is required to achieve a cosmetic effect. Therefore, the cosmetic applications of SLNs are the most thoroughly studied. The development of cosmetic applications relies on the aforementioned advantages and the shorter time to market for cosmetics than for drug products, which is due to the regulatory steps that must be addressed in the pharmaceutical field and to safety and efficacy failures during clinical trials [5]. Particular interest is reserved for moisturizing and anti-aging cosmetics [2]. In fact, due to their moisturizing properties, SLNs can themselves improve skin elasticity and, as such, can be further used to load anti-aging antioxidant ingredients, for whom they can promote skin permeation [1].

Several methods for the preparation of SLNs have been described in the literature, with the most promising being those that are solvent-free and easy to scale up, such as the hot homogenization process [6]. Nonetheless, further research can be implemented to find reproducible methods to formulate SLNs with improved technological and biological features, such as reduced particle size, which seems to be a key point for enhancing the cosmetic performance of SLNs on the skin. To this aim, this experimental work has optimized a patented, reproducible, easy to scale-up and solvent-free technology in order to formulate ultrasmall (<100 nm) SLNs using several lipid matrixes and biocompatible ingredients. In preliminary experiments, 6-coumarin (6-cum) was used as the model compound for loading into SLNs. An SLN formulation was then loaded with α-tocopherol (TOCO). The anti-aging properties of the TOCO SLNs were studied on cell models and on porcine ear-skin models for cutaneous permeation, taking advantage of 6-cum fluorescent labeling.

## 2. Experimental

### 2.1. Materials

#### 2.1.1. Chemicals

Tripalmitin (Tp), trimystirin (Tm) and cholesteryl palmitate (Cp) were obtained from Fluka (Buchs, Switzerland). Polysorbate 20–Tween 20 (Tw20) was obtained from A.C.E.F spa (Piacenza, Italy). Sorbitan esters–Span (Sp), Sp40, Sp60, and Sp80 were obtained from Rol Oil (Alessandria, Italy); 80% hydrolyzed polyvinyl alcohol of 9000–10,000 MW (PVA9000), hexyl hexadecanoate (cetylpalmitate) (Hh), Sepharose CL 4B, 6-cum, 60,000–90,000 MW dextran, dimethyl sulfoxide (DMSO), sodium dodecyl sulfate (SDS) and methanol (MeOH) were obtained from Sigma-Aldrich (St. Louis, MO, USA). Sodium chloride (NaCl) was obtained from Merck. TOCO was obtained from Hoffman (Basilea, Switzerland). Eagle’s minimal essential medium (EMEM), Fetal Bovine Serum (FBS), penicillin and streptomycin (100 units/mL), and hyaluronic acid 800 kDa (HA) were provided by Altergon S.r.l. Distilled water was purified using a MilliQ system (Millipore, Bedford, MO, USA).

#### 2.1.2. Cells

Assays on human RBC were performed on 20 µL samples of finger-tip capillary blood, which was collected from the operator in a sterile laboratory setting. Primary human dermal fibroblasts (HDF, purchased from Lonza) were cultured at passage 5–6 with complete medium, composed of Eagle’s minimal essential medium (EMEM) supplemented with 20% FBS, 100 U/mL penicillin, 100 U/mL streptomycin and 2X non-essential amino-acids. The HDF cells were maintained in 100 mm-diameter cell-culture dishes in a humidified and controlled atmosphere at 37 °C and 5% CO_2_. The medium was changed every 3–4 days.

#### 2.1.3. Tissues

Thin slices of porcine skin, with a thickness of 1 mm, were obtained from pig ears using an Acculan^®^ dermatome (Aesculap, Tuttlingen, Germany) and then frozen at −18 °C. Before the in vitro permeation studies, the skin was thawed and equilibrated for 30 min in saline solution (NaCl 0.9% *w*/*v*) that was supplemented with sodium azide (0.01% *w*/*v*) to preserve the skin [7].

### 2.2. Methods

#### 2.2.1. SLN Formulation

The SLNs were prepared using the Polysorbate Sorbitan Phase-Inversion Temperature (PS-PIT) technique [8]. Three ingredients were employed: a solid lipid, a water-in-oil (W/O) emulsifier and a polyoxythilene-based surfactant. Four different lipids were investigated as alternative solid lipids: two triglycerides (Tp and Tm), and two esters (Cp and Hh). Tw20 was used as the polyoxythilene-based surfactant, whereas three different Sp were compared for use as W/O emulsifiers: Sp40, Sp60, and Sp80. The SLNs were obtained in a single vial following multiple heating and cooling cycles. Briefly, the operating conditions were as follows: the solid lipid, W/O emulsifier and Tw20 were heated in distilled water under stirring; as the temperature increased, the mixture became clear because of microemulsion formation, and it then turned turbid because of phase inversion.

During cooling the same steps took place in reverse order: from being turbid, the mixture became clear, and then turbid again when the microemulsion collapsed into a nanoemulsion. Finally, the SLN precipitation occurred when the temperature fell below the lipid-melting point. Heating/cooling cycles were repeated several times in order to reduce the mean SLN size.

#### 2.2.2. SLN Purification

The SLNs were purified from the hemotoxic Tw20 excess [9] using either size-exclusion chromatography (Sepharose CL 4B) or 30% dextran-gradient centrifugation and resuspension. In the first case, 1 mL of SLNs was eluted in a Sepharose CL 4B column with NaCl solution. The fractions collected were either turbid (purified SLNs) or clear (Tw20 micelles).

In the second case, 0.5 mL of SLNs were diluted with 0.5 mL of 60,000–90,000 MW dextran 30% water solution, followed by centrifugation at 25,000 rpm for 10 min (Allegra^®^ 64 R centrifuge, Beckmann Coulter, Palo Alto, CA, USA). The supernatant, containing Tw20 micelles, was removed, whereas the lipid pellet (purified SLNs) was resuspended in 0.5 mL of distilled water.

#### 2.2.3. Phase-Transition Study

Temperature-dependent phase transitions were studied using a conductivity meter (Orion Research, Conductivity meter mod 101, Texas City, TX, USA).

#### 2.2.4. Compound Loading in SLNs

SLNs were loaded with TOCO, an antioxidant agent, and 6-cum, a lipophilic fluorescent probe, both alone and in combination. TOCO, being a lipid-like substance, was added directly to the SLNs (SLN TOCO-8 mg/mL), whereas 6-cum was pre-dissolved in DMSO and then added to the SLNs (SLN 6-cum-0.1 or 0.4 mg/mL). In both cases, the compounds were loaded into SLNs at the phase-inversion temperature.

#### 2.2.5. SLN Characterization

The mean particle sizes (intended as hydrodynamic diameter) and polydispersity of SLNs were determined using the dynamic light-scattering technique (DLS; 90 Plus, Brookhaven Instrument Corporation, Holtsville, NY, USA). Size measurements, obtained with three replicates at an angle of 90° at a temperature of 25 °C, are shown as mean ± SEM. Statistical analysis was performed owing to the two-tailed *t*-test: *p*-Value < 0.005 was considered significant. The homogeneity of the suspension was checked using optical microscopy (DM2500, Leica Microsystems, Wetzlar, Germany).

Differential scanning calorimetry (DSC) was performed on the SLNs using a DSC 7 (Perkin-Elmer, Waltham, MA, USA). The bulk lipid materials, polyoxythilene-based surfactants and SLN suspensions were purified by dextran-gradient centrifugation and resuspension and were compared. Each dried sample was placed in a conventional aluminum pan and heated from 30 °C to 80 °C at 2 °C min^−1^. The SLN degree of crystallinity (% crystallinity degree) was estimated by calculating the ratio between the melting enthalpy/g lipid of the SLN dispersions and the melting enthalpy/g of the lipid bulk material [10,11].

SLN morphology was determined using Transmission Electronic Microscopy (TEM, FEI Tecnai G12 Spirit Twin, Eindhoven, The Netherlands) and Field Emission Scanning Electron Microscopy (FE-SEM, Tescan, S9000G, Brno, Czech Republic). In the case of TEM, a LaB_6_ emission source (120 kV, spotsize 1) and 400 mesh carbon-coated copper grids were used at room temperature (RT). The carbon-coated copper grid was immersed in ultra-diluted nanoparticle suspensions and, after the drying step, the grid was placed on a rod holder for TEM characterization.

Three grids were prepared per NP suspension and a minimum of four micrographs were acquired per grid. For FE-SEM, the samples were metalized with a 5 nm chromium layer before analysis. 6-cum % recovery and % entrapment efficiency (EE%) were determined by spectrophotometric analysis. 6-cum % recovery was calculated as the ratio between the total amount of probe in the SLNs vs. the total weighed 6-cum. The 6-cum EE% was calculated as the ratio between the amount of probes in purified SLN (either by size exclusion or 30% dextran-gradient centrifugation and resuspension) vs. the total amount of probes in the SLNs. The TOCO % recovery was calculated via HPLC analysis, while TOCO EE% was not determined, being a lipid-like compound.

#### 2.2.6. RBC Scratch

An RBC scratch test was performed to ascertain the efficiency of SLN purification from the Tw20 excess. The SLNs that were purified by size exclusion or 30% dextran-gradient centrifugation and resuspension were tested. In the first case, purified SLNs were used as such, while, in the second case, purified SLNs were diluted with 0.1, 0.2 and 0.3 mL of NaCl solution. Five microliters of either human or mouse blood and 5 µL of purified SLNs were mixed together on a microscope slide, and the RBC scratch was performed with another slide, placed at 45° from the surface support. RBC morphology was checked using optical microscopy (DM2500, Leica Microsystems, Wetzlar, Germany). An 8% Tw20 solution and a nanoemulsion for total parenteral nutrition (Intralipid^®^) were used as positive and negative toxicity controls, respectively.

#### 2.2.7. Wound-Healing Assay

In order to evaluate any hypothetical effect that the TOCO SLNs may have on wound repair, a wound-healing assay was carried out on HDF. The following samples were evaluated:

- negative control: EMEM with 2% of FBS, penicillin and streptomycin (100 units/mL), essential amino acids;
- positive control: HA 1% in EMEM;
- TOCO SLNs dispersed in EMEM at 1% *v*/*v*;
- HA/TOCO SLNs: TOCO SLNs dispersed in 1% HA solution.

HDF cells were cultured in 100 nm Petri dishes. Once the HDF reached confluence (~60–70%), they were subjected to a senescence protocol via UV irradiation (UV lamp 254–365 nm VL6 230 nm 50/60 Hz) for 4 days. UV irradiation was applied at 4000 J/m^2^ for 5.50 min, twice a day.

For the scratch assay, senescent and non-senescent HDF (1 × 10^3^ cell/mL for well) were seeded in 96-well plates in triplicate up to 24 h. Afterward, the HDF monolayer was scraped in a straight line to create a “scratch” with a p10 pipet tip. The debris was removed by washing the cells once with 1 mL of the growth medium, and the medium was then replaced with 2 mL of culture medium that is specific for this in vitro assay.

This assay medium is composed of a lower percentage of FBS (2%) than used in the growth medium; this is to minimize cell proliferation while being sufficient to prevent apoptosis and/or cell detachment. Samples were incubated with damaged HDF at 37 °C in a 5% CO_2_ humidified atmosphere. Wound-healing images were acquired immediately after the scratch, at 0 h (A_t=0_), and at 3, 7 and 24 h (A_t_), in quadruplicate using bright-field microscopy. Wound area was estimated using ImageJ public domain software in a region of interest (ROI), and % wound closure, directly related to HDF migration, was calculated as follows:

$$\mathrm{Wound}\;\mathrm{Closure}\;\%=\frac{A_{t=0}-{\;A}_{t}}{A_{t=0}}\times \;100$$

where A_t=0_ is the area of the wound measured immediately after the scratch and A_t_ is the area of the wound measured at various instants of time after the execution of the scratch. The percentage of closure clearly increases as the cells migrate toward the scratch over time. All the experiments were performed at least in three parallel groups with three replicates per sample, and the results are shown as mean ± SD. Data analysis was performed using GraphPad software. The repeated results were compared with the ordinary two-way analysis of variance ANOVA (Tukey’s multiple comparison test) and a *p*-value < 0.0001 was considered significant.

#### 2.2.8. Skin-Permeation Studies

SLN permeation studies were carried out on pig-ear skin according to the Beck and Bracher protocol [12]. Thin layers of pig-ear skin were placed on a vertical Franz diffusion cell, with the compartments held together by a clamp. An appropriate aliquot (2 mL) of SLNs Hh 2%, Sp80 2%, Tw20 4% co-loaded with TOCO and 6-CUM, purified via size exclusion, was applied to the skin surface to completely and uniformly cover the stratum corneum, with a diffusion area of 1.60 cm^2^. Free TOCO/6-cum extemporary suspension was used as the control. Briefly, known amounts of TOCO and 6-cum were co-dissolved in methanol under mild heating; then methanol was evaporated with a vacuum evaporator centrifuge (Hetovac VR-1, De Mari Strumenti, Milan, Italy) and the obtained 6-cum solution in liquid TOCO was dispersed in 1% PVA9000 water solution under sonication (Transsonic, Elma Schmidbauer GmbH, Singen, Germany). The receptor medium, consisting of saline solution (NaCl 0.9% *w*/*v*) containing SDS (0.1% *w*/*v*), used as a solubilizing agent, was continuously mixed using a magnetic stirring bar over the entire measurement period [13,14].

Proper aliquots were withdrawn at regular time intervals (1–24 h) for the HPLC analysis of TOCO and 6-cum, and the cell was immediately refilled with fresh receptor solution. At the end of the experiment (24 h), the application site on the skin was washed with tap water to remove the residual formulation from the surface, which was tape-stripped. Subsequently, the skin was cut into small pieces with a scalpel. TOCO and 6-cum were extracted from the tape-stripped and crumbled skin using MeOH (15 mL and 5 mL, respectively) under stirring for 6 h, in order to be quantified via HPLC analysis. Experiments were performed five times and the results were reported as mean ± SEM. Statistical analysis was performed using Prism GraphPad 5.0 (San Diego, CA, USA) and using Student’s *t*-test.

In separate experiments at the end of the permeation studies, the skin was cut using a cryostat (Reichert-Jung/Leica, Frigocut 2800 N, Deer Park, IL, USA), giving 5 μm sections that were used to observe 6-cum permeation via fluorescence optical microscopy λ_exc_ = 450 nm, λ_em_ = 490 nm (DM2500, Leica Microsystems, Wetzlar, Germany).

#### 2.2.9. 6-cum Spectrophotometric Analysis

In the case of the SLNs purified by size exclusion, 0.1 mL of the purified SLN suspension and 0.4 mL of Tw20 micelles were diluted with 0.4 mL MeOH. Both were centrifuged for 5 min at 14,000 rpm (Allegra^®^ 64 R centrifuge, Beckmann Coulter, Palo Alto, CA, USA). The supernatant was analyzed spectrophotometrically (λ = 450 nm, Perkin-Elmer, Lambda 2 UV/VIS Spectrophotometer, Waltham, MA, USA). In the case of the SLNs purified by dextran-gradient centrifugation, 0.2 mL of supernatant that was isolated from centrifugation was diluted with 0.3 mL MeOH, while 0.1 mL purified SLNs were suspended in 0.4 mL MeOH. Both suspensions were centrifuged for 5 min at 25,000 rpm and analyzed spectrophotometrically (λ = 450 nm).

#### 2.2.10. HPLC Analysis

HPLC analysis was employed to determine TOCO % recovery in SLNs and for skin-permeation studies. Briefly, in order to determine the compounds loaded, 3 µL of the SLNs purified by size-exclusion chromatography were diluted in 3 mL of MeOH, followed by centrifugation at 14,000 rpm for 5 min. Franz cells receiving phases and skin extractions were injected as such. HPLC analysis was performed with a YL9110 Quaternary Pump, equipped with a YL 9160 diode array detector (PDA-Yang Lin, Anyang, Republic of Korea) and a Shimadzu RF-20 fluorescence detector (Shimadzu, Tokyo, Japan), linked to Clarity software for data analysis (Yang Lin, Anyang, Republic of Korea). The column was a C8 Lichrosphere 100 µm 4.6 mm × 8 cm (Sigma Aldrich, St. Louis, MO, USA). The PDA detector was set at λ = 290 nm for TOCO and λ = 450 nm for 6-cum. The fluorimeter for 6-cum was set at excitation λ_exc_ = 450 nm and emission λ_em_ = 490 nm. The flow rate was set at 1 mL/min, with the eluent gradient below (Table 1).

The 6-cum retention time was 4.0 min, while the TOCO retention time was 7.5 min.

## 3. Results

### 3.1. Formulation Screening and Characterization of SLNs

Different types of solid lipids (Tp, Tm, Cp, Hh) and different W/O emulsifiers Sp (Sp40, Sp60, Sp80) were used for SLN preparation in the screening study. Tw20 was employed as the polyoxythilene-based surfactant. In particular, the following three combinations were evaluated:

- 2% lipid, 4% Sp, 8% Tw20;
- 2% lipid, 2% Sp, 5% Tw20;
- 2% lipid, 4% Sp, 5% Tw20.

Sp60 was employed as the W/O emulsifier for all types of solid-lipid matrixes; in the case of Hh, Sp40 and Sp80 were also investigated. For the combination 2% lipid 4% Sp 8% Tw20 SLN, formations occurred for all types of solid lipids used, whereas, for the combination 2% lipid 2% Sp 5% Tw20, SLNs failed to form when Sp40 was employed as the W/O emulsifier. For the combination of 2% lipid 4% Sp 5% Tw20, SLNs only formed when Hh and Sp80 were used (Table 2). Therefore, Hh and Sp80 were selected as the most versatile ingredients for SLN preparation.

**Figure 1:**
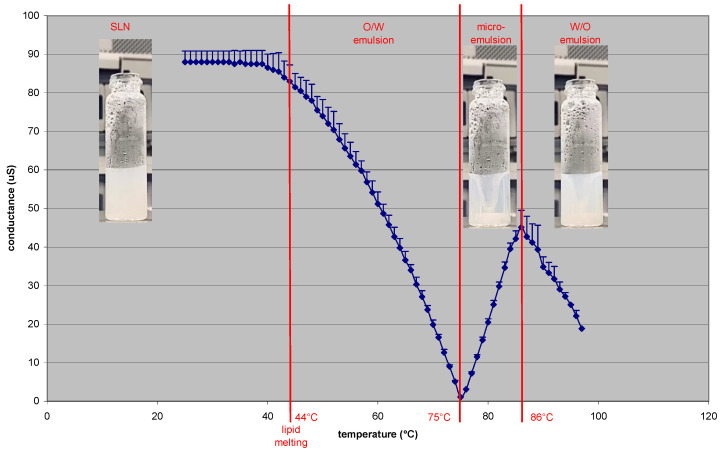
Conductivity variations due to temperature in the Polysorbate-Sorbitan Phase Inversion Temperature (PS-PIT) process.

The temperature-dependent phase transitions observed in the PS-PIT process (see Supplementary Video S1) were studied using a conductometer. Analysis was carried out on non-purified SLNs 2%Tm 4% Sp60 8% Tw20 (Figure 1).

It is worth noting that SLN conductivity decreases as the temperature increases; from 90 µS at room temperature, it lowers starting from the solid-lipid melting point (T_onset_ = 44 °C), because of the formation of the O/W emulsion. This may be attributed to the shift of Tw20, which is in excess in the SLN outer phase, to the O/W interface during emulsion formation. When the temperature reaches 75 °C (minimum conductivity of 1 µS), the O/W emulsion collapses because of the formation of the microemulsion (clear phase). By this point, the temperature rises together with conductivity until 50 µS. This probably occurs because Tw20 quickly shifts between droplets through the outer phase when it is in a microemulsion, unlike when it is in an O/W emulsion, resulting in increased conductivity. When the temperature rises above the PIT (nearly 86 °C), Tw20 becomes water insoluble and phase inversion occurs [15]; the mixture turns turbid and conductivity rapidly decreases to 20 µS. However, in view of the high W/O ratio, it is improbable that a real W/O emulsion is formed. Indeed, a multiple W/O/W emulsion is likely to be formed, in which W/O-rich portions are dispersed in water.

DSC analyses were performed in order to compare the melting points and enthalpies of the different blank SLNs. The acquired thermograms of the lipid bulk materials (Tp, Tm, Cp, Hh), polyoxyethylene-based surfactants (Sp40, Sp60) and blank SLNs are reported in Table 3 and Figure S1. Sp80 was not considered because it is a liquid at room temperature.

The melting point is depressed when the lipid bulk-matrix material is turned into SLNs [16], and the presence of impurities, surfactants and stabilizers may also affect this phenomenon [17,18]. Indeed, the melting-point decrease of SLNs may be due to the colloidal dimensions of the particles, in particular to their high surface-to-volume ratio, and not to the recrystallization of the lipid matrices in a metastable polymorph [10]. It should be noted that the melting-point shift is lower for SLNs with a high lipid/Sp ratio than it is for SLNs with a low lipid/Sp ratio. However, all of the SLNs considered show a single transition peak above room temperature, which corresponds to a single colloidal species in suspension, with the absence of polymorphism and “supercooled melts”. Moreover, Sp causes the lowering of both the lipid melting point and enthalpy in proportion to its concentration. Therefore, an Sp excess makes SLNs more amorphous.

In preliminary experiments, 6-cum was loaded into SLNs (0.1 mg/mL) and was used as the model compound. Both blank SLNs and 6-cum loaded SLNs were characterized from a physico-chemical standpoint, after purification with size-exclusion. In the case of 6-cum loaded SLNs, dextran-gradient centrifugation and resuspension was employed as a purification method for comparison purposes. Complete particle-size characterization is shown in Table 4.

Among SLNs purified by size exclusion, 6-cum-loaded SLNs are smaller than blank SLNs (40–250 nm vs. 70–700 nm), except in the case of SLNs 2% Hh 4% Sp80 8% Tw20, with many statistically significant differences reported. In particular, when Hh is employed as the solid-lipid matrix, 6-cum loaded SLNs mean size ranges between 40 and 200 nm, and several formulations are <100 nm (ultrasmall SLNs), with interesting physicochemical and biological properties to be obtained. Moreover, differences in 6-cum SLNs size can be observed according to the purification technique employed. Indeed, SLNs show higher size after purification by dextran-gradient centrifugation and resuspension than by size exclusion, with several formulations showing statistically significant differences. This is probably due to the irreversible particle aggregation that occurs during the centrifugation step. On the other hand, 6-cum EE% was higher when the SLNs were purified by dextran-gradient centrifugation and resuspension and reached almost 90% in the case of SLNs 2% Hh 4% Sp80 8% Tw20. Therefore, we can state that Hh and Sp80 delivered the best compromise between particle-size reduction and EE%, provided that drug loading can reduce particle size in a relevant manner, and that size exclusion avoids aggregation during purification.

**Figure 2:**
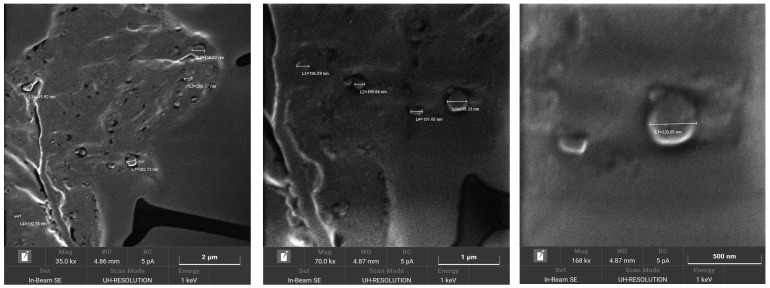
Field Emission Scanning Electronic Microscopy (FE-SEM) characterization of 6-coumarin (6-cum)-loaded SLNs 2% Hh 2% Sp80 5% Tw20. Abbreviations: Hh: hexadecyl hexadecanoate (cetylpalmitate); Sp80: Span 80; Tw20: Tween 20.

6-cum-loaded SLN morphology was investigated using FE-SEM after purification by size-exclusion chromatography. In Figure 2 SLNs 2% Hh 2% Sp80 5% Tw20, which displayed a spherical shape, are shown as an example.

Finally, in order to ascertain the efficiency of SLN purification from the hemotoxic Tw20 excess [9], an RBC scratch test was performed on human RBC. The formulations tested were: 6-cum SLNs 2% Hh 4% Sp80 8% Tw20, purified by size exclusion (130 nm); 6-cum SLNs 2% Hh 2% Sp80 5% Tw20, purified by size exclusion (220 nm), and; 6-cum SLNs 2% Hh 2% Sp80 5% Tw20, purified by dextran-gradient centrifugation and resuspension (240 nm). An 8% Tw20 solution and a nanoemulsion for total parenteral nutrition (Intralipid^®^) were used as positive and negative toxicity controls, respectively. As can be seen in Figure S2, purified SLNs showed no hemotoxicity, regardless of the purification method used and their particle size. Indeed, unlike 8% Tw20, no morphological alterations were observed either in human and mouse RBC in the case of the SLNs, or in the case of Intralipid^®^, whose biocompatibility has been assessed by decades of clinical use.

### 3.2. Formulation and Characterization of TOCO SLNs

**Figure 3:**
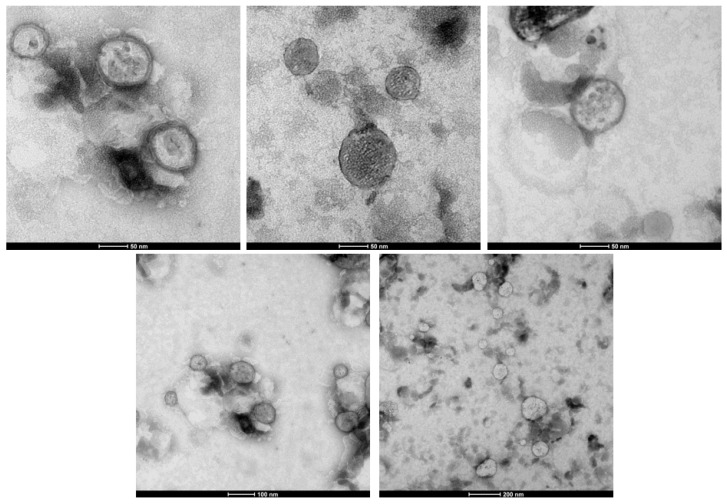
Transmission Electronic Microscopy (TEM) of α-tocopherol (TOCO) loaded solid lipid nanoparticles (SLNs).

Based on the results obtained from the formulation screening, suitable SLN prototypes were optimized for the loading of the antioxidant agent TOCO. Indeed, due to their ultrasmall size and lipid-like nature, SLNs can promote TOCO permeation to the deep dermis, where it can act on the cells responsible for skin aging. Specifically, Sp80 and Hh were chosen as the W/O emulsifier and lipid matrix, respectively, because in the above-mentioned studies, they maximized the EE% of the model compound 6-cum and minimized mean particle size. The following ingredient ratio was chosen for the formulation of TOCO SLNs: 2%Hh 2% Sp80 4%Tw20 0.8% TOCO. TOCO SLNs were purified by size exclusion and underwent sterile filtration (GVS, Bologna, Italy) in order to be employed in cell studies for anti-aging activity. Furthermore, TOCO SLNs were also labeled with 6-cum (0.4 mg/mL) in order to allow fluorometric detection in skin-permeation studies. The physicochemical characterization of SLNs is reported in Table 5, while TEM characterization is reported in Figure 3.

As shown in Table 4, these SLN formulations can be defined as ultrasmall because they display a mean size below 100 nm. In fact, images acquired using TEM (Figure 3) show SLNs with a spherical shape and mean size of 90 nm. As previously observed for 6-cum in the formulation screening, the loading of exogenous compounds into the SLNs also leads to reductions in the mean particle size in the case of TOCO but does not consistently affect the polydispersion index.

### 3.3. Wound-Healing Assay

**Figure 4:**
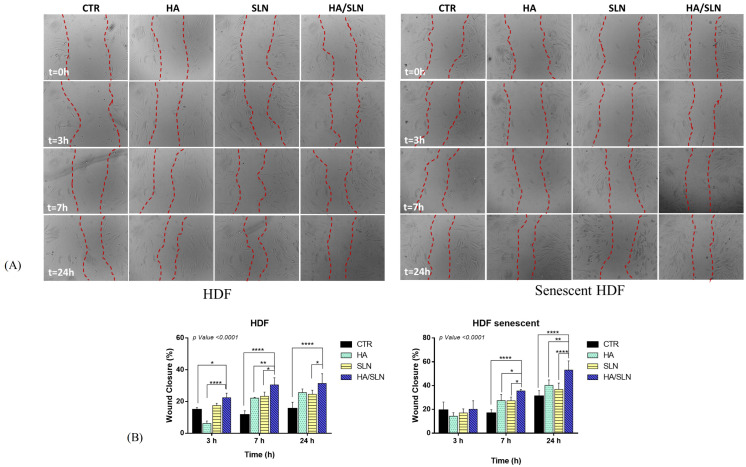
(**A**) Representative Bright-field images obtained by Optical Microscopy of senescent and non-senescent Human dermal fibroblasts (HDF) after scrape, upon incubation with the formulations. Definition of region of interest (ROI–red line) for % wound closure definition. (**B**) % wound closure after 3, 7, 24 h of incubation with formulations. Abbreviations: CTR: control; HA: hyaluronic acid; HDF: human derived fibroblasts; SLN: solid lipid nanoparticles. All results are presented as mean ± standard deviation. For HDF experimental set: * *p* value < 0.0001 vs. SLN, **** *p* value < 0.0001 vs. CTR (24 h); * *p* value < 0.0001 vs. SLN, ** *p* value < 0.0001 vs. HA, **** *p* value < 0.0001 vs. CTR (7 h); **** *p* value < 0.0001 vs. HA; * *p* value < 0.0001 vs. CTR (3 h). For senescent HDF experimental set: **** *p* value < 0.0001 vs. SLN, ** *p* value < 0.0001 vs. HA, **** *p* value < 0.0001 vs. CTR (24 h); * *p* value < 0.0001 vs. SLN, * *p* value < 0.0001 vs. HA, **** *p* value < 0.0001 vs. CTR (7 h).

In order to evaluate the anti-age effect of SLNs Hh 2% Sp80 2% Tw20 4% loaded with TOCO, an in vitro Scratch Assay was performed on senescent and non-senescent HDF. Untreated cells were the negative control; HA was used as the positive control. Images of wound healing were acquired after incubating the scraped HDF monolayers with the formulations for 0, 3, 7 and 24 h (Figure 4). A reduction in scrape area can be observed from 0 to 24 h in both senescent and non-senescent HDF for all formulations tested.

This trend was confirmed when comparing % wound closure, calculated as described in Section 2.2. In the case of TOCO SLNs, the % wound closure was higher than negative controls for all exposure times, both on senescent and non-senescent HDF, whereas it was lower than HA on senescent HDF. However, the highest % wound closure was detected when HDF were incubated with the HA/SLNs combination, and this is probably because of the synergic wound-healing action of HA and the TOCO SLNs.

### 3.4. Skin-Permeation Studies

**Figure 5:**
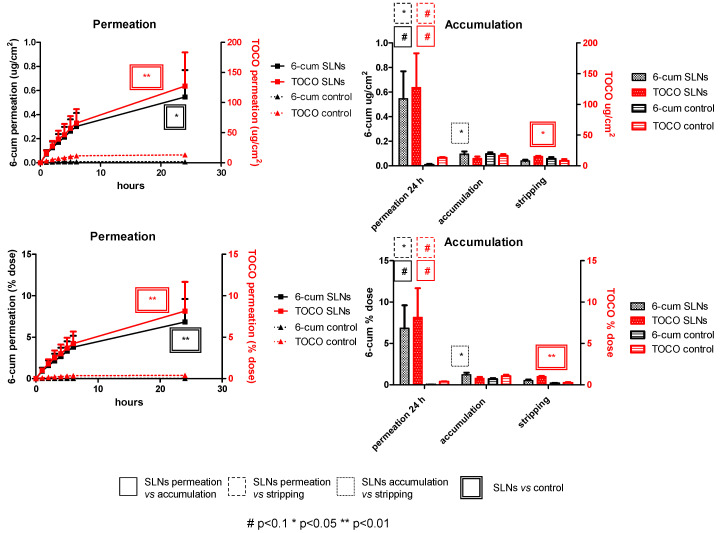
Permeation, accumulation and tape-stripping, through pig-ear skin, of 6-coumarin (6-cum) and α-tocopherol (TOCO) co-loaded solid lipid nanoparticles (SLNs).

Permeation studies were carried out on pig-ear skin. To this aim, TOCO SLNs were fluorescently labeled with 6-cum to better elucidate tissue distribution and mimic the co-loading of additional cosmetic ingredients. TOCO and 6-cum skin permeation and accumulation were determined by HPLC analyses. As shown in Figure 5, the TOCO and 6-cum permeation patterns are similar, with a higher total dose permeating through the skin at 24 h than that accumulated in the skin or recovered by stratum corneum tape-stripping.

Subsequently, histological investigations were carried out on cryo-sectioned skin in separate experiments (Figure S3). 6-cum fluorescence was mainly localized on the surface of the stratum corneum of the skin. However, in some sections, 6-cum was observed to have diffused into the deeper skin layers, indicating that SLNs may favor the skin permeation of loaded compounds.

## 4. Discussion

Using the PS-PIT technique, SLNs can be obtained in a solvent-free, economical and scalable process with low energy consumption. Several kinds of solid lipids, such as triglycerides, aliphatic and cholesteryl esters, were employed in the matrixes, allowing spherical-shape and solid-state nanoparticles to be produced. The loading of 6-cum, used as the model compound, into PS-PIT SLNs causes a significant decrease in mean particle size, which falls within the ultrasmall range (<100 nm). Excess polysorbates can be removed either via ultracentrifugation and resuspension, or size exclusion. The latter method is preferable as it avoids particle aggregation. Ultrasmall SLNs display innovative technological and biological features, allowing their prospective use in biomedical applications. Indeed, they can be sterilized via filtration and have the potential to overcome biological barriers.

However, as previously mentioned, cosmetic applications have the shortest time to market as they can exploit the ability of SLNs to enhance the permeation of loaded compounds by means of skin hydration and adhesion. Reduced particle size plays a key role in such mechanisms. In fact, according to recent studies, a monolayer can theoretically be formed by applying approximately 4 mg of formulation/cm^2^ of 4% SLNs onto the skin, and this monolayer can exert an occlusive action by slowing the moisture loss caused by evaporation. Nonetheless, different occlusion degrees can be achieved, according to particle size. An occlusion factor of only 10% has been experimentally observed for lipid microparticles (diameter > 1 μm), whereas that figure is 50% for lipid 200 nm nanoparticles. In fact, the dimensions of the air channels between nanoparticles are much smaller than those in microparticles, and this provides an improved occlusion effect [1]. SLNs with reduced particle size can, therefore, be exploited to increase the skin permeation of anti-age cosmetic ingredients that act in the dermis. For example, ultrasmall SLNs (approximately 85 nm sized) containing 10% coenzyme Q10 demonstrated a greater free-radical-scavenging effect after exposure to UV light than conventional SLNs and nanoemulsions. The surfactant used to stabilize SLNs is also relevant; quercetin-loaded SLNs, stabilized with polysorbates, have demonstrated an increased hydration effect along with polysorbate concentrations of up to 2%. Finally, due to its synergism with the moisturizing effects of SLNs, TOCO has been suggested for use as a preferential anti-aging agent to be loaded into SLNs [19,20].

Accordingly, research evidence matches the current trends in marketed cosmetics. In 2005, Cutanova Cream NanoRepair Q10 (Dr. Rimpler GmbH, Wedemark, Germany) was the first SLN-based anti-aging cosmetic product to be introduced onto the market. It was an SLN-containing cream that was superior, in terms of skin hydration, to a conventional cream. NanoLipid Restore CLR (Chemisches Laboratorium Dr. Kurt Richter, Berlin, Germany) is a semi-finished SLN-based cosmetic product. Upon loading into SLNs, black currant seed oil and the anti-age coenzyme Q10 are protected from oxidation, improving the stability of the final product. In the Surmer cosmetic product line (Dr. Rimpler GmbH, Wedemark, Germany), SLNs have been used to increase the occlusion of a day cream without changing its light character, i.e., without causing the skin to have the shiny appearance that is associated with conventional high-occlusion night creams [3].

The formulation approach employed for TOCO SLNs in this experimental work takes into account the most relevant indications from the previously mentioned research and market benchmarks. Indeed, their most innovative technological features include (1) the ultra-small dimensions, which are reported to favor skin adhesion and occlusion; (2) the fairly high-melting Hh lipid matrix, which augments occlusive properties; (3) the presence of polysorbate surfactants as the stabilizers, with the excess surfactant being removed by molecular exclusion and the residue acting as a permeation promoter for the loaded functional ingredients; (4) the advantageous use of TOCO as an anti-aging agent, which, due to its lipidic nature, can be loaded into SLNs at high concentration; (5) the possibility of co-delivering different functional ingredients, such as the model fluorescent compound 6-cum, using the same SLNs. SLN-loaded TOCO and 6-cum showed high skin permeation in vitro, although remarkable experimental variability was observed. Follicular absorption can also be hypothesized in addition to the aforementioned mechanisms. In fact, the presence of hair follicles on the skin models adopted for this experiment is not homogeneous, and this may account for the variation in the obtained experimental evidence [21]. Due to its low solubility, TOCO has been extensively loaded in lipid-based cosmetic and/or dermatological formulations [22,23,24,25,26,27,28,29]. According to recent literature, ultrasmall PIT SLNs allow a relevant skin permeation, vs. both skin accumulation from the same SLNs and permeation from control suspension. However, few literature reports account for pure TOCO skin permeation [30], because ingredients employed to solubilize it can act themselves as skin permeation enhancers, owing to several mechanisms.

Finally, HA, used in our experimental studies as a reference, can also provide a technological advantage, besides its potential anti-aging action [31]. In fact, SLN suspensions are too poorly viscous for skin application, and various strategies have, therefore, been proposed for the preparation of semisolid systems: (1) the dispersion of SLN suspensions in conventional semisolid formulations (e.g., gels, creams and ointments); (2) the direct addition of gelling agents to SLN suspensions, with a high concentration of lipids. The second strategy is more interesting because it avoids the excessive dilution of the loaded functional ingredients [2]. In this case, HA can be used to increase the viscosity of SLN suspensions in order to obtain serum for cosmetic use. Furthermore, it is well known that HA leads to an enhancement in cell migration, allowing the formation of soft tissue with good elasticity, and increased microvascular density [32,33]. In particular, the healing process may be explained by HA action during the re-epithelialization phase of the wound-healing process. HA activates the proliferation and migration of keratinocytes and fibroblasts and promotes dermal collagen remodeling during morphogenesis [34]. Therefore, given the promising results here reported, TOCO SLNs with HA can be proposed as a cosmetic semisolid formulation with an anti-aging activity that can be further functionalized with other cosmetic ingredients.

## 5. Conclusions

SLNs, made up of triglycerides, aliphatic and cholesteryl esters, can be obtained via the solvent-free, economical and scalable PS-PIT method with low energy consumption. The loading of compounds into the SLN lipid matrix leads to particle-size reduction within the ultrasmall range, with interesting technological and biological properties. Given that the cosmetic application of SLNs has the shortest time to market, ultrasmall TOCO SLNs show several advantages for anti-age cosmetics as they can favor the skin absorption of loaded compounds through the stratum corneum.

## 6. Patents

WO2020/254934 A1.

## Figures and Tables

**Table 1:** HPLC gradient.

Time (min)	% Water	% MeOH
0	60	40
5	0	100
10	0	100
11	60	40

**Table 2:** Composition and labeling of blank 2% solid lipid nanoparticles (SLNs) used in pre-formulation studies. Abbreviations: Cp: cholesteryl palmitate; Hh: hexadecyl hexadecanoate (cetylpalmitate); Sp: Span; Tm: Trimyristin; Tw: Tween.

Lipid (2%)	Sp Grade	Sp and Tw Ratio		
		4% Sp 4, 8% Tw20%	2% Sp, 5% Tw20	4% Sp, 5% Tw20
Tp	Sp60	SLNs 2% Tp 4% Sp60 8% Tw20	SLNs 2% Tp 2% Sp60 5% Tw20	-
Tm	Sp60	SLNs 2% Tm 4% Sp60 8% Tw20	SLNs 2% Tm 2% Sp60 5% Tw20	-
Cp	Sp60	SLNs 2% Cp 4% Sp60 8% Tw20	SLNs 2% Cp 2% Sp60 5% Tw20	-
Hh	Sp60	SLNs 2% Hh 4% Sp60 8% Tw20	SLNs 2% Hh 2% Sp60 5% Tw20	-
	Sp40	SLNs 2% Hh 4% Sp40 8% Tw20	-	-
	Sp80	SLNs 2% Hh 4% Sp80 8% Tw20	SLNs 2% Hh 2% Sp80 5% Tw20	SLNs 2% Hh 4% Sp80 5% Tw20

**Table 3:** Differential scanning calorimetry (DSC) parameters (T_onset_, T_peak_ ΔH_fus_). Abbreviations: Cp: cholesteryl palmitate; Hh: hexadecyl hexadecanoate (cetylpalmitate); SLNs: solid lipid nanoparticles; Sp: Span; Tm: Trimyristin; Tw: Tween.

	T_onset_	T_peak_	ΔH_fus_ (J/g)
Tp	61.16 °C	63.58 °C	166.86
Sp60	53.48 °C	58.18 °C	69.54
SLNs 2% Tp 2% Sp60 5% Tw20	58.28 °C	61.81 °C	16.84
SLNs 2% Tp 4% Sp60 8% Tw20	46.02 °C	51.94 °C	29.31
Tm	59.56 °C	60.18 °C	207.35
SLNs 2% Tm 2% Sp60 5% Tw20	55.90 °C	56.61 °C	31.53
SLNs 2% Tm 4% Sp60 8% Tw20	44.01 °C	52.01 °C	18.53
Cp	76.60 °C	77.58 °C	70.00
SLNs 2% Cp 2% Sp60 5% Tw20	73.94 °C	75.43 °C	15.57
SLNs Cp 2% Sp60 4% Tw20 8%	66.25 °C	71.71 °C	4.10
Hh	53.44 °C	54.34 °C	206.05
Sp40	43.59 °C	47.44 °C	66.76
SLNs 2% Hh 4% Sp40 8% Tw20	49.23 °C	52.51 °C	21.41
SLNs 2% Hh 4% Sp60 8% Tw20	50.53 °C	51.14 °C	4.33
SLNs 2% Hh 4% Sp80 8% Tw20	52.61 °C	54.21 °C	44.77

**Table 4:** Dynamic Light Scattering (DLS) particle size characterization of blank and 6-cum loaded solid lipid nanoparticles (SLNs) purified by either size exclusion chromatography or dextran-gradient centrifugation and resuspension. Abbreviations: 6-cum: 6-coumarin; Cp: cholesteryl palmitate; Hh: hexyl hexadecanoate; N.D.: not determined (SLNs not formed or exceeding DLS detection capability): PDI: polydispersion index; SLNs: solid lipid nanoparticles; Sp: span; Tm: trimystirin; Tp: tripalmitin; Tw20: tween 20. Statistical analysis: 6-cum SLNs purified by size exclusion vs. blank SLNs purified by size exclusion *p* < 0.005 *; *p* < 0.001 **; *p* < 0.0005 ***; *p* < 0.0001 ****. 6-cum SLNs purified by dextran-gradient centrifugation and resuspension vs. 6-cum SLNs purified by size exclusion *p* < 0.0005 ^§§§^; *p* < 0.0001 ^§§§§^.

SLNs	Blank SLNs Purified by Size Exclusion		6-cum SLNs Purified by Size Exclusion		6-cum SLNs Purified by Dextran-Gradient Centrifugation and Resuspension	
	Mean Size (nm)	PDI	Mean Size (nm)	PDI	Mean Size (nm)	PDI
2% Tp 4% Sp60 8% Tw20	711.4 ± 50.8	0.327	255.9 ± 7.1 **	0.293	293.9 ± 6.2	0.256
2% Tp 2% Sp60 5% Tw20	N.D.	N.D.	168.5 ± 3.3	0.237	139.8 ± 35.9	0.281
2% Tm 4% Sp60 8% Tw20	202.2 ± 4.0	0.159	75.1 ± 2.0 ****	0.255	219.7 ± 6.3 ^§§§§^	0.248
2% Tm 2% Sp60 5%Tw20	N.D.	N.D.	170.1 ± 0.1	0.184	469.3 ± 138.4	0.284
2% Cp 4% Sp60 8% Tw20	210.8 ± 4.6	0.204	64.4 ± 0.9 ****	0.228	213.2 ± 3.2 ^§§§§^	0.219
2% Cp 2% Sp60 5% Tw20	579.3 ± 26.6	0.228	236.0 ± 3.4 *	0.220	347.5 ± 82.1	0.203
2% Hh 4% Sp60 8% Tw20	71.9 ± 0.9	0.153	46.8 ± 1.8 ***	0.309	145.6 ± 36.7	0.463
2% Hh 2% Sp60 5% Tw20	315.8 ± 4.1	0.088	98.8 ± 1.8 ****	0.251	122.8 ± 2.9	0.290
2% Hh 4% Sp40 8% Tw20	100.5 ± 0.8	0.132	56.6 ± 0.5 ****	0.219	190.3 ± 3.1 ^§§§§^	0.331
2% Hh 4% Sp80 8% Tw20	71.2 ± 3.6 ****	0.266	132.7 ± 1.5	0.184	233.7 ± 6.7 ^§§§^	0.257
2% Hh 2% Sp80 5% Tw20	296.1 ± 0.9	0.151	192.6 ± 1.7 ****	0.197	210.9 ± 15.4	0.252
2% Hh 4% Sp80 5% Tw20	91.0 ± 1.5	0.127	84.1 ± 1.1	0.251	250.2 ± 8.4 ^§§§§^	0.291

**Table 5:** Physicochemical characterization of blank (2% hexadecyl hexadecanoate-Hh, 2% Span80-Sp80, 4% Tween20-Tw20), α-tocopherol (TOCO) loaded and 6-coumarin (6-cum) and TOCO co-loaded solid lipid nanoparticles (SLNs) purified by size exclusion. Abbreviations: PDI: polydispersion Index.

	Mean Size (nm)	PDI	Loaded Compound Concentration after Purification (μg/mL)
Blank SLNs	297.2 ± 4.1	0.185	-
TOCO SLNs	108.6 ± 0.8	0.162	2250 ± 250
6-cum TOCO SLNs	97.0 ± 0.1	0.102	TOCO: 2990 ± 304 6-cum: 15.2 ± 2.3

## Data Availability

The data presented in this study are available on request from the corresponding author.

## Supplementary Materials

The following supporting information can be downloaded at: https://www.mdpi.com/article/10.3390/pharmaceutics15071962/s1, Figure S1. DSC Thermograms. (A) Red: Tp; black: Sp60; blue: SLNs 2% Tp, 2% Sp60, 5% Tw20; green: SLNs 2% Tp, 4%Sp60, 8% Tw20. (B) Red: Tm; black: Sp60; blue: SLNs 2% Tm, 2% Sp60, 5% Tw20; green: SLNs 2% Tm, 4% Sp60, 8% Tw20. (C) Red: Cp; black: Sp60; blue: SLNs 2% Cp, 2% Sp60, 5% Tw20; green: SLNs 2% Cp, 4% Sp60, 8% Tw20. (D) Red: Hh; black: Sp60; blue: SLNs 2% Hh, 2% Sp60, 5% Tw20; green: SLNs 2% Hh, 4% Sp60, 8%Tw20. (E) Red: Hh; pink: Sp40; black: Sp60; light blue: SLNs 2% Hh, 4% Sp40, 8% Tw20; blue: SLNs 2% Hh, 4% Sp60, 8% Tw20; green: SLNs 2% Hh, 4% Sp80, 8% Tw20. Abbreviations: Cp: cholesteryl palmitate; Hh: hexadecyl hexadecanoate (cetylpalmitate); Sp: Span; Tm: Trimyristin; Tw: Tween. Figure S2. RBC scratch for different SLNs formulations. Magnification 630×. Abbreviations: 6-COU: 6-coumarin; Hh: hexyl hexadecanoate; PDI: polydispersion index; SLNs: solid lipid nanoparticles; Sp: span; Tw20: tween 20. Figure S3. Fluorescent and optical microscopy of cryo-sectioned skin after 24 h. Magnification 630×. Supplementary Video S1.
