# Supplementary material for: Ultrasmall Solid-Lipid Nanoparticles via the Polysorbate Sorbitan Phase-Inversion Temperature Technique: A Promising Vehicle for Antioxidant Delivery into the Skin
Source: Pharmaceutics. 2023 Jul 16;15(7):1962. doi: 10.3390/pharmaceutics15071962 (PMC10383899; doi:10.3390/pharmaceutics15071962)
Supplement: Supplementary file 1 [file pharmaceutics-15-01962-s001.zip › pharmaceutics-2452554-supplementary.pdf]

Supplementary Materials

Ultrasmall Solid Lipid Nanoparticles by the Polysorbate Sorbitan Phase Inversion Temperature: a promising vehicle for antioxidant delivery into the skin

Francesca Della Sala, Assunta Borzacchiello, Chiara Dianzani, Elisabetta Muntoni, Monica Argenziano, Maria Teresa Capucchio, Maria Carmen Valsania, Annalisa Bozza, Sara Garelli, Maria Di Muro, Franco Scorziello, Luigi Battaglia

Figure S1 page S2

Figure S2 page S3

Figure S3 page S5

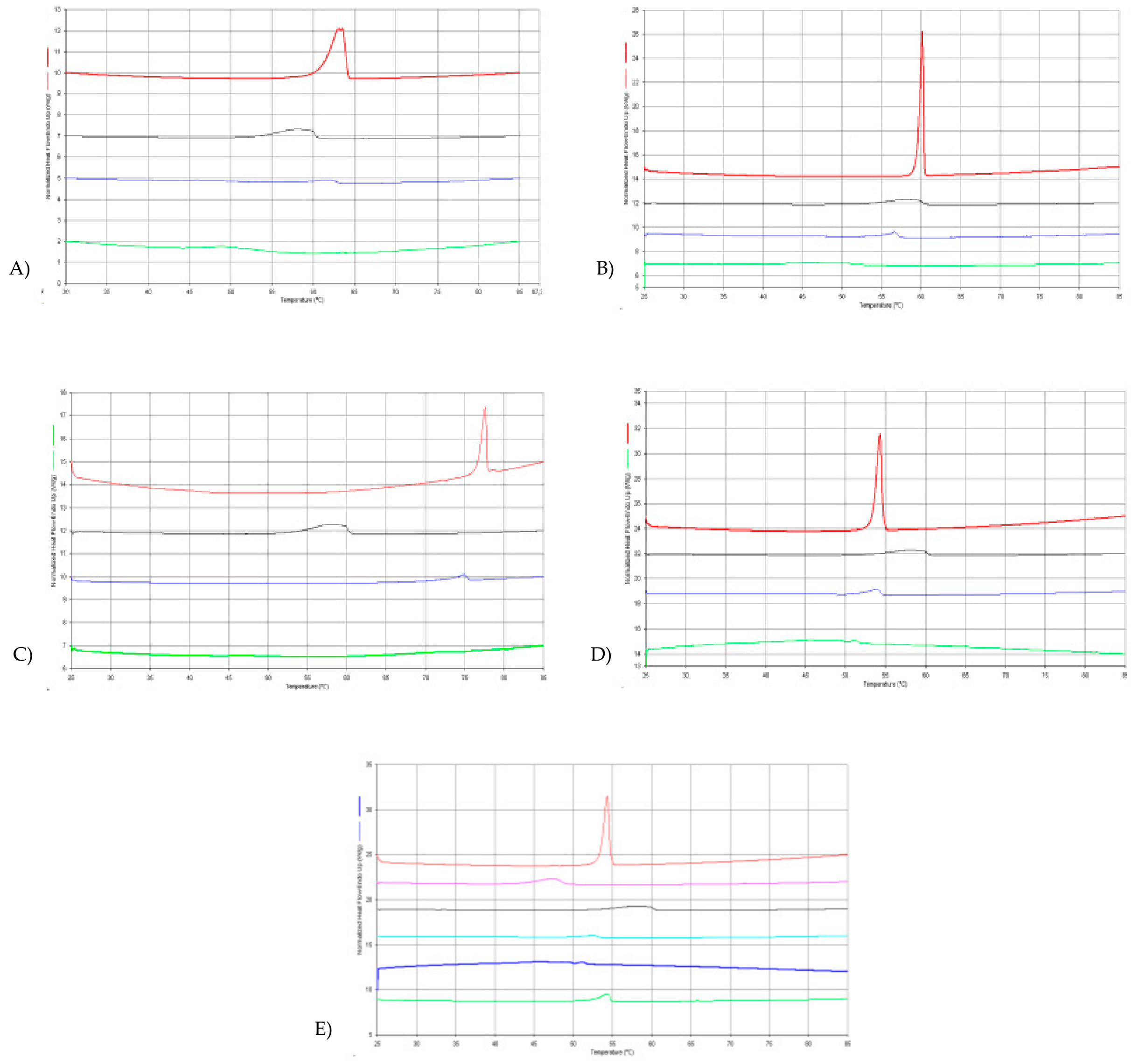

Figure S1. DSC Thermograms. A) Red: Tp; black: Sp60; blue: SLNs 2% Tp, 2% Sp60, 5% Tw20; green: SLNs 2% Tp, 4%Sp60, 8% Tw20. B) Red: Tm; black: Sp60; blue: SLNs 2% Tm, 2% Sp60, 5% Tw20; green: SLNs 2% Tm, 4% Sp60, 8% Tw20. C) Red: Cp; black: Sp60; blue: SLNs 2% Cp, 2% Sp60, 5% Tw20; green: SLNs 2% Cp, 4% Sp60, 8% Tw20. D) Red: Hh; black: Sp60; blue: SLNs 2% Hh, 2% Sp60, 5% Tw20; green: SLNs 2% Hh, 4% Sp60, 8%Tw20. E) Red: Hh; pink: Sp40; black: Sp60; light blue: SLNs 2% Hh, 4% Sp40, 8% Tw20; blue: SLNs 2% Hh, 4% Sp60, 8% Tw20; green: SLNs 2% Hh, 4% Sp80, 8% Tw20. Abbreviations: Cp: cholesteryl palmitate; Hh: hexadecyl hexadecanoate (cetylpalmitate); Sp: Span; Tm: Trimyristin; Tw: Tween

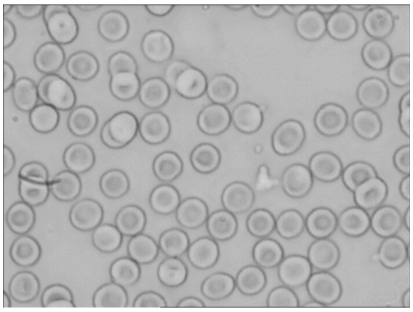
NaCl solution (blank)

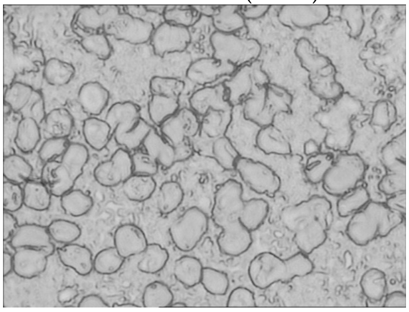
8% Tw20 (negative control)

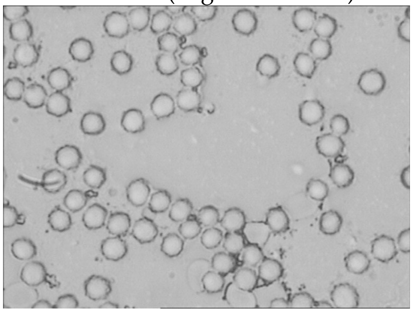
Intralipid® (positive control)

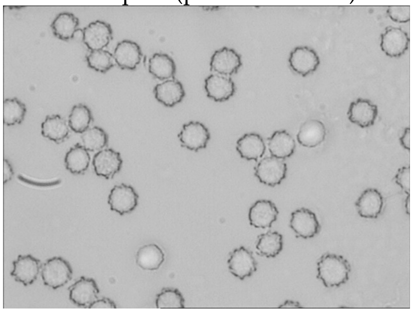
6-COU loaded SLNs 2% Hh 4% Sp80 8% Tw20 purified by size exclusion

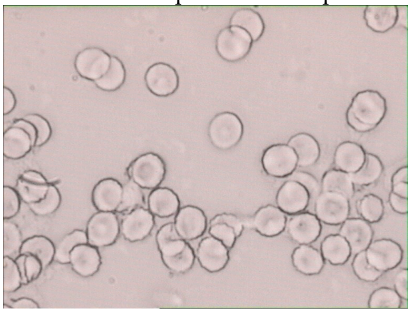
6-COU loaded SLNs 2% Hh 2% Sp80 5% Tw20 purified by size exclusion

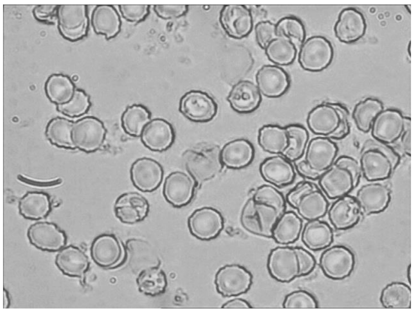

6-COU loaded SLNs 2% Hh 2% Sp80 5% Tw20 purified by dextran-gradient centrifugation & resuspension (diluted in 100 µL NaCl)

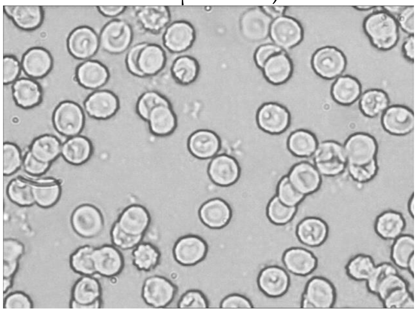

6-COU loaded SLNs 2% Hh 2% Sp80 5% Tw20 purified by dextran-gradient centrifugation & resuspension (diluted in 200 µL NaCl)

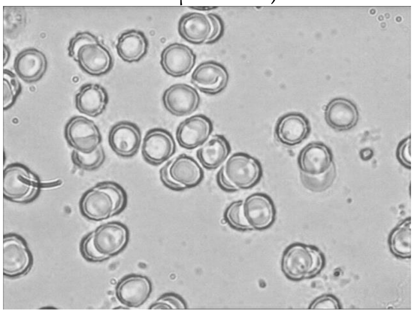

6-COU loaded SLNs 2% Hh 2% Sp80 5% Tw20 purified by dextran-gradient centrifugation & resuspension (diluted in 300 µL NaCl)

Figure S2. RBC scratch for different SLNs formulations. Magnification 630x. Abbreviations: 6-COU: 6-coumarin; Hh: hexyl hexadecanoate; PDI: polydispersion index; SLNs: solid lipid nanoparticles; Sp: span; Tw20: tween 20.

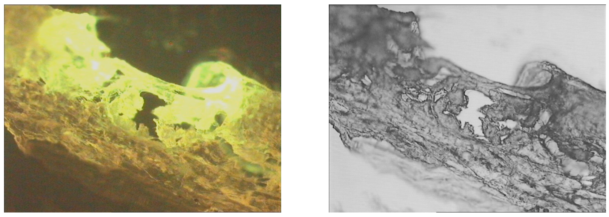

Figure S3. Fluorescent and optical microscopy of cryo-sectioned skin after 24h. Magnification 630x
